# Nutrient-Induced Changes of Liver Fat Content in Humans

**DOI:** 10.33549/physiolres.935525

**Published:** 2025-04-01

**Authors:** Jan KOVÁŘ, Rudolf POLEDNE

**Affiliations:** 1 Institute for Clinical and Experimental Medicine, Prague

**Keywords:** Metabolic-dysfunction associated steatotic liver disease (MASLD), Non-alcoholic fatty liver disease (NAFLD), Triglyceride, Hepatic fat content (HFC), Dietary intervention

## Abstract

Metabolic-dysfunction associated steatotic liver disease (MASLD) affects approximately 30 % of the world adult population and even contributes to the increased mortality from cardiovascular disease. Dietary intervention, along with exercise, is the most important tool for the treatment of MASLD patients. Dietary composition can have profound effects on liver fat. This review summarizes the results of studies that used MR methods to study the effect of macronutrients on liver fat content. It focuses on intervention studies manipulating the content and quantity of macronutrients in long-term dietary intervention studies and, in more detail, on studies monitoring the effect of administered nutrients on changes in liver fat over several hours.

## Metabolic-dysfunction associated steatotic liver disease (MASLD)

Metabolic-dysfunction associated steatotic liver disease (MASLD) [1], formerly referred to as non-alcoholic fatty liver disease (NAFLD) [2–4] is currently one of the most widespread diseases. It affects approximately 30 % of the world adult population [5,6] and because of its principal characteristic, accumulation of fat in the liver that is not easy to detect, remains undiagnosed in most of the patients. The disease begins as simple steatosis, which apparently does not cause any health problems, but which can progress to steatohepatitis in some patients. In some of these patients, it can progress further to cirrhosis and hepatocellular carcinoma [7]. The disease is even becoming the leading cause of liver transplantation in developed countries [8]. On the other hand, only handful of patients with MASLD progress to this stage of liver disease; most of the patients with MASLD die from cardiovascular disease and recent data even indicated MASLD as an independent risk factor of cardiovascular disease [1].

The pandemics of MASLD seems to be closely related to pandemics of obesity, metabolic syndrome and diabetes. MASLD is even considered a hepatic manifestation of metabolic syndrome and some authors are even convinced that this is its first manifestation [9]. The disease affects up to 75 % of obese patients [10] and 65 % of patients with type 2 diabetes [11].

Most importantly, the pathological accumulation of fat in the liver appears to have lipotoxic effect inducing endoplasmic reticulum stress, mitochondrial dysfunction and lysosomal dysfunction [12]. It likely represents a crucial role in progression from relatively benign simple steatosis to metabolic-dysfunction associated steatohepatitis (MASH). The inflammatory response of the liver can induce stellate cells activation and fibrogenesis which already represents a serious clinical problem.

## Triglyceride metabolism in the liver

The simple steatosis (metabolic-associated fatty liver (MAFL)) - the first step in pathogenesis of MASLD - results from imbalance between acquisition of liver fat and its oxidation or export from the liver in very low density lipoproteins (VLDL). There are three principal sources of liver fat – dietary fat, fatty acids released from adipose tissue in the process of lipolysis, and de novo lipogenesis.

Dietary fat enters circulation in chylomicrons and most of chylomicron triglycerides (TG) is hydrolyzed in capillaries of extrahepatic tissues by the action of lipoprotein lipase (LPL) [13]. The fatty acids of dietary origin then enter the liver via two routes – as part of chylomicron remnants, which are taken up by the liver via LDL-receptor related protein (LRP) and LDL-receptor, and as fatty acids released from chylomicrons by LPL but not taken up by extrahepatic tissues. These are referred to as “spillover” fatty acids. Approximately 20 % of dietary fat is transported to the liver through these pathways [7]. It can be estimated that at least 10 g of dietary fat can enter the liver daily.

However, most of the liver fat is synthesized from fatty acids released during lipolysis of triglycerides in adipose tissue and transported into circulation. Lipolysis in adipose tissue is suppressed by insulin and, therefore, the transport of fatty acids from adipose tissue to the liver takes place in postabsorptive phase. Fatty acids originating from adipose tissue make up almost 60 % of hepatic fat [14].

The third source of liver fat, the synthesis of fatty acids from non-lipid precursors – carbohydrates and aminoacids in de novo lipogenesis seem to be of minor importance in healthy subjects. However, increased de novo lipogenesis seems to play a critical role in pathogenesis of MASLD [15].

The fatty acids of liver triglycerides can be mobilized, transported to mitochondria and enter β-oxidation. The acetyl-CoA produced can be then oxidized to produce ATP in citrate cycle or serve as a substrate for production of ketone bodies that are used as energy substrate for other tissues. It is estimated that 30 to 60 g of ketone bodies is synthesized in the liver daily even in subjects who are not fasting [16].

Last but not least, liver triglycerides can be mobilized and secreted into circulation in VLDL to provide their fatty acids to other tissues. The daily production of VLDL-TG is on the order of 25 g but may significantly vary between individuals – it has been demonstrated that it positively correlates with liver fat content [17].

Several gene variants have been identified that play a role in the development of an imbalance between the accumulation and utilization of liver fat [18]. Two of them, which are of greatest importance, are directly involved in lipid metabolism. Variant I148M of patatin-like phospholipase domain-containing 3 protein encoded by PNPLA3 gene does participate in suppressing the lipolysis of triglycerides in lipid droplets, thereby reducing the ability of hepatocytes to mobilize fat for export in VLDL [19]. Variant E1678K of transmembrane 6 superfamily member 2 protein encoded by TM6SF2 gene does not properly fulfill its role in VLDL assembly and is associated with defective VLDL secretion [20]. Both these variants apparently reduce the ability of hepatocytes to eliminate excess liver fat. Both variants are also associated with an increased risk of disease progression to inflammation and fibrosis [21,22]. Importantly, it has been shown that the development of MASLD in carriers of defective PNPLA3 variant is independent of the development of insulin resistance [23]. Another variant - P446L of glucokinase regulator encoded by GCKR gene does not effectively mediate inhibition of glucokinase by fructose-6-phospate and in this way contributes to the increased influx of glucose into de novo lipogenesis [24].

## Quantification of liver fat

A key problem in the study of fat accumulation in the liver has long been the fact that no tool was available to quantify the liver fat content. Liver biopsy is the gold standard method for diagnosing MASLD, but its use for quantifying liver fat is arguable because the question how representative is the tissue sample taken. Neither sonographic methods nor different biochemical scores do not allow the quantification of hepatic fat. This problem was solved only after the pioneering study by Longo *et al*. [25], who were the first to introduce proton magnetic resonance (MR) spectroscopy into the determination of liver fat content. Using this method, a normal range of hepatic fat content (HFC) could be defined by the Dallas Heart Study group [26]. The distribution of HFC was found to be skewed in 345 subjects without any MASLD risk factors, with a median concentration of 1.9 % and upper limit of normal values of 5.5 %. Healthy human subjects with a median HFC may then have approximately 25 g of triglycerides in their liver.

## The role of nutrients in development of MASLD

The main cause of current pandemic of obesity, insulin resistance, type 2 diabetes and also MASLD is a modern lifestyle associated with excessive energy intake and reduced physical activity. The increased consumption of fat and added sugars mainly contributes to increased energy intake. These nutrients not only provide calories, but also act as important regulatory signals that can affect hepatic lipid metabolism. Fatty acids, especially n-3 polyunsaturated fatty acids (PUFAs), serve as ligands for nuclear peroxisome proliferator-activated receptors (PPARs). The activation of PPARα and PPARβ/δ that are expressed in the liver results in increased uptake and beta-oxidation of fatty acids [27]. The glucose consumption stimulate insulin secretion and insulin then promotes de novo lipogenesis through activation of sterol regulatory element-binding protein 1c (SREBP1c) metabolic pathway. Moreover, metabolites arising in the metabolism of glucose and fructose in the liver interact with another nuclear receptor – carbohydrate-responsive element-binding protein (ChREBP), which also stimulates de novo lipogenesis [28].

This review will aim to summarize the results of studies that used MR methods to study the effect of macronutrients on liver fat content. We will focus primarily on intervention studies manipulating the content and quantity of macronutrients both in long-term dietary intervention studies (lasting from a few days to 2 years) and also in studies monitoring the effect of administered nutrients on changes in liver fat over several hours.

## Dietary intervention-induced changes of liver fat content

A large number of studies have attempted to find out how the consumption of various macronutrients affects the accumulation of fat in the liver. When comparing the results of these studies, it is necessary to take into consideration that the reduction of one of the diet components is necessarily compensated by an increase of another one – if we reduce the proportion of carbohydrate in the diet, the proportion of fat will increase. Importantly, caloric intake is another important player in that context and it has been repeatedly documented that the changes in liver fat content are fundamentally determined by the amount of calories consumed [29]. Therefore, we will analyze the results of long-term dietary intervention studies according to whether energy intake was higher, lower or the same as energy expenditure (hypercaloric, hypocaloric and isocaloric diets, respectively).

## Changes in liver fat content induced by long-term hypercaloric diets

Several studies analyzed the impact of different nutrients using a hypercaloric feeding regimen. In these studies subjects usually received a specified dose of the nutrient being studied, which they had to consume in addition to their normal diet.

Some studies have looked at the effects of increased fat intake on HFC. Feeding ten young, lean men with a high-fat diet (extra 675 kcal per day mainly in dairy fat) for 4 days resulted in 90 % increase in liver fat [30]. Very similar results, 86 % increase in liver fat, were obtained in a study of 30 young lean men who received high-fat diet containing abundant 35 % energy as saturated fat for a week [31]. The accumulation of fat in the liver was observed in 17 lean subjects consuming the diet enriched with saturated fatty acids for 7 weeks (in caloric excess of 750 kcal per day), but not in the group of 18 subjects consuming diet enriched with polyunsaturated fatty acids [32]. Importantly, weight gain did not differ between groups.

Due to the association between the intake of added sugars, particularly fructose, and the incidence of MASLD coming out from the observational studies [33,34], most of the studies focused on the role of extra energy provided by simple carbohydrates.

In earlier study in which 7 healthy lean volunteers consumed fructose in excess of 1.5 g/kg of body weight daily for 4 weeks no effect on body weight and hepatic fat was observed [35]. In another study from the same group 24 healthy lean males consumed hyper-caloric high-fructose diet (3.5 g of fructose/kg of fat free mass) for 7 days and such treatment resulted in significant increase in weight and almost 80 % increase in liver fat [36]. When added sugars were used in caloric excess of 1000 kcal a day for 3 weeks in 16 overweight men, body weight and liver fat increased by 2 % and 17 %, respectively [37]. The similar results were found in a study carried out in 10 overweight subjects (6 males, 4 females) that were drinking 1 liter of regular Coca Cola daily (106 g of sugar) for 6 months – hepatic fat significantly increased [38]. Several studies compared the effect of the same amount of fructose and glucose providing an excess energy. In a study of 32 centrally obese overweight men, consumption of glucose and fructose in excess of 25 % of total energy for 2 weeks resulted in increase of both weight and liver fat and the response was same to both sugars [39]. The similar results were obtained in a study of 20 subjects consuming 3.5 g of fructose or glucose/kg of fat free mass for a week [40]. On the contrary, no effect on HFC was found in a study on 20 subjects receiving 150 g of glucose or fructose daily in addition to a balanced weight-maintaining diet for 4 weeks [41].

When protein in excess of 450 kcal per day was added to a hypercaloric high-fat diet fed to subjects for 4 days, accumulation of hepatic fat was reduced compared to a high fat-diet despite an increase in body weight [30]. Similarly, in a study in 6 healthy lean males fed for 6 days a hypercaloric high-fructose diet (3 g of fructose/kg of weight), the accumulation of liver fat was blunted by supplementing the diet with 24 g of essential amino acids a day [42].

To summarize, excessive intake both saturated fat and carbohydrate that leads to an increase in weight results also in accumulation of liver fat. Administration of polyunsaturated fat results also in body weight increase but hepatic fat is not affected. If glucose and fructose are administered in excess, there are no differences in the response of body weight and hepatic fat to both sugars. Last but not least, addition of protein to the hypercaloric diet protects the liver against the accumulation of fat.

## Changes in liver fat content induced by long-term hypocaloric diets

A number of intervention studies investigated the effect of hypocaloric diets on changes in liver fat. It should be noted that most of these studies were carried out in obese or overweight subjects and they intended to significantly reduce body weight.

In two studies in which morbidly obese patients [43] or patients with type 2 diabetes [44] were treated with a very low-calorie diet (450–800 kcal/day) for several weeks, it was found that along with a significant weight loss by about 15 %, there was also a decrease in liver fat content by 43 to 80 %.

A beneficial effect on liver fat was also observed in a number of other studies that used more moderate caloric restriction but lasted for a several months to 2 years [37,45–47].

Two long-term studies comparing the effects of diets with a low and high fat content (respectively high and low carbohydrate content) found no difference in their beneficial effects on liver fat [47,48]. In contrast, a two-week intervention with a low-carbohydrate diet reduced liver fat more than a low-calorie diet with restricted amount of fat [49]. Interestingly, only a 6-day ketogenic diet also resulted in a profound decrease in hepatic fat; the magnitude of decrease was affected by the PNPLA3 polymorphism [50,51].

It can be concluded that administration of hypocaloric diets that successfully reduce body weight leads to a significant decrease in liver fat content regardless of the nutritional composition of the diet used. These findings strongly support a role of weight reduction in the treatment of MASLD.

## Changes in liver fat content induced by long-term isocaloric diets

Studies using isocaloric diets are probably the most important for understanding the role of macronutrients in inducing changes in liver fat, as they are not associated with changes in body weight. However, when interpreting the results of these studies, it should be kept in mind that if the proportion of one of the nutrients changes, the proportion of the other nutrients will also change.

Studies that addressed the issue of increased fat intake nicely documented that the administration of a high-fat diet containing approx. 55 % of total energy as fat for 2 to 6 weeks induces an increase in HFC compared to a low-fat diet [52–54]. Importantly, the effect of saturated fatty acids (SFAs) and n-6 PUFAs on the liver fat was compared in a 10-week study carried out in 67 abdominally obese subjects. The HFC was 16 % lower on n-6 PUFA diet than on SFA diet [55]. Similar results were obtained in a study carried out in patients with type 2 diabetes that compared the effect of 8-week intervention using diets rich in monounsaturated fatty acids (MUFAs) and that rich in carbohydrate and fiber. While feeding a MUFA-rich diet resulted in a decrease in liver fat, a high-carbohydrate diet had no effect on HFC [56].

Importantly, some studies also compared the effect of different carbohydrates. Replacing part of the complex carbohydrates in the diet by fructose (25 % of energy content) in a small study in 8 healthy men for 9 days led to an increase in hepatic fat that could be explained by increased de novo lipogenesis as measured using stable isotopes [57]. In a similar study in obese children with metabolic syndrome, fructose restriction to 4 % of energy content resulted in a significant decrease of liver fat [58]. On the other hand, another study that compared the effect of consumption of glucose- and fructose-sweetened beverages on hepatic fat in adolescents found no difference in the effect of both sugars on liver fat content, despite improved insulin sensitivity in the glucose group [59]. Similarly, in a 2-week study in 32 healthy overweight men, no difference in the effect of diets containing fructose and sucrose (25 % of energy content) on hepatic fat was observed [39]. Moreover, hepatic fat was also unaffected in a study where subjects consumed varying amounts of dietary fructose (as sucrose or high-fructose corn sirup) for 10 weeks [60].

To summarize, increased intake of dietary fat results in accumulation of liver fat on isocaloric diet. On the other hand, the evidence is not so strong to support the role of fructose in the development of hepatosteatosis.

## Nutrient-induced acute changes of liver fat content

The intervention studies discussed above lasted usually from a few days up to a few months and provided some guidance how to modify dietary recommendations in patients with MASLD.

Considering the quantity of fat that can be delivered into the liver each day from both the intestine and the adipose tissue or synthesized from non-lipid precursors, the daily turnover of TG exceeds their content in the liver, at least in healthy subjects with normal fat content. Therefore, it can be anticipated that HFC can fluctuate reasonably during the day and that changes in HFC induced by nutrient intake can be detected by MR even within a few hours. Only a limited number of studies have analyzed how the administration of different nutrients affects liver fat over the course of only a few hours and their results are summarized in Table 1.

First, it should be pointed out that short-term fasting for 12 hours has no effect on HFC as documented in the study in eight healthy men [61]; the changes in HFC induced by fasting were observed only after a minimum of 48 hours of fasting [61,62].

The first study that tried to determine whether HFC can be affected nutrient administration was seminal study by Dallas Heart Study group [26]. The authors measured HFC after overnight fast and 4 hours after high-fat mixed meal containing 50 g of fat in 7 subjects with HFC less than 3 % and one subject with steatosis. Although plasma TG increased to double 4 hours after meal, HFC was not affected.

In another study young lean healthy volunteers (6 males, 3 females) received high-fat meal containing 61.5 % energy as fat and equaling 50 % of daily recommended intake (80 ± 12 g of fat, 92 ±14 g of carbohydrate, and 20 ± 3 g of protein) [63]. Meal administration led to an expected increase in insulinemia, plasma TG, and suppression of non-esterified fatty acid (NEFA) concentration. Plasma glucose did not change significantly during the study. HFC content rose from 3.1 ± 0.9 to 3.7 ± 0.7 % 3 hours after meal and did not change further for another 2 hours. The addition of 51 ± 8 g of protein to the meal induced a more pronounced response of insulin and TG concentrations, bud had no effect on response of HFC which rose from 2.9 ± 0.3 to 3.7 ± 0.4 % after 4 hours and remained unchanged after 5 hours. This strongly suggests that addition of protein has no significant acute effect on fat accumulation in the liver.

Bilet *et al*. [64] did not find any significant effect of 2-hour cycling exercise on liver fat content. However, ingesting glucose (1.4 g/kg of body weight) before the exercise prevented fat accumulation in the liver during post-exercise recovery compared with an experiment in which the same subjects drank water. The authors explained their findings by suppression of fatty acid flux from the adipose tissue to the liver - NEFA concentration was indeed suppressed after glucose and increased after water administration.

Hernández *et al*. [65], who studied the effect of pure fat administration (palm oil, 1.8 g/kg of body weight) on HFC in 14 young and lean healthy male volunteers, observed that HFC rose by 35 % from 0.93 ± 0.26 to 1.26 ± 0.32 % 4 hours after meal. Administration of pure fat resulted in expected change of plasma TG, but insulin concentration was not affected and NEFA tended to rise during experiment. Using hyperinsulinemic euglycemic clamp carried out 6 hours after palm oil administration, they noted that insulin sensitivity was decreased.

In our study [66], ten non-obese subjects with normal HFC were given 150 g of fat in a cream and HFC measured before and 3 and 6 hours after administration. The HFC in these subjects rose by 19 % from 1.99 ± 1.28 to 2.25 ± 1.34 % six hours after meal. In another study in eight non-obese subjects with hepatosteatosis using exactly the same design [67] HFC rose by 10 % from 11.9 ± 7.8 to 13.1 ± 8.6 %. Converted to the total amount of newly deposited fat, the subjects with steatosis accumulated almost five times more fat than those with normal liver fat. There is currently no explanation for such findings. It cannot be excluded that the livers of these patients uptake more fatty acids from circulation - it has been documented that MASLD patients have upregulated hepatic fatty acid transclocase CD36 [68]. We also noted that allelic frequency of PNPLA3 and TM6SF2 gene variants associated with increased MASLD risk due to impaired export of TG from the liver in VLDL was severalfold increased in patients with steatosis (0.28 vs. 0.05) [66,67].

To study the effects of glucose and/or fructose administration on acute changes of liver fat we administered these sugars three times during 6-hour lasting examinations [66,67,69]. In this way we were able to ensure that NEFA concentration was suppressed throughout the entire examination. Both glucose and fructose efficiently suppress the flow of NEFA from adipose tissue to the liver, which allows minimizing their role in liver fat accumulation.

Administration of 3 x 50 g of fructose at time 0, 2, and 4 hours had no effect on HFC in both non-obese subjects with normal fat content and with hepatosteatosis [66,67] and also in obese subjects with MASLD [69]. No effect on accumulation of fat in the liver could be observed even if the fructose was co-administered with high-fat load.

On the other hand, administration of 3 x 50 g of glucose at time 0, 2, and 4 hours resulted in decrease of HFC by 15 % from 1.73 ± 0.83 to 1.47 ± 0.75 % after six hours in non-obese subjects with normal fat content [66]. Moreover, the co-administration of glucose with high-fat load prevented accumulation of liver fat after high-fat load. However, when the same examinations were carried out in both non-obese subjects and obese subjects with MASLD, administration of glucose alone had no effect on accumulation of liver fat [67,69]. When the glucose was co-administered with high-fat load to the non-obese subjects with MASLD, HFC rose transiently from 13.8 ± 8.2 to 14.8 ± 7.8 % three hours after meal and then returned to the baseline (14.1 ± 7.8 %).

## Conclusions

In summary, long-term dietary intervention studies clearly documented that both hypercaloric and hypocaloric interventions that induce body weight change also induce the change of liver fat that are positively correlated and that such an effect is independent of nutrients used.

Increased intake of dietary fat appears to be associated with liver fat accumulation in both hypercaloric and isocaloric long-term intervention studies, although this effect can be attenuated with increased content of unsaturated fat (both as MUFA and PUFA). These results are in a full agreement with the results of studies of acute changes after a high-fat load. This suggests that the liver does not seem to properly deal with the overload of dietary fat. The amount of dietary fat required to elicit a response of liver fat detectable by MR methods in acute experiments cannot be determined exactly, but it seems that 1 g of fat/kg of body weight appears to be sufficient. Incidentally, the fat administered to the subjects in these studies was predominantly saturated and it is not clear yet whether an increased proportion of MUFA or PUFA in the fat load can suppress the accumulation of fat in the liver in acute experiments. Interestingly, recent preclinical studies suggest that providing n-3 PUFAs in phospholipids rather than triglycerides may be even more effective in mediating their effect on the liver fat [70].

The role of dietary carbohydrates in affecting changes of hepatic fat is more complex. Although the results of most long-term intervention studies support the role of simple sugars in the pathogenesis of MASLD, some contradictions regarding the role of glucose and fructose still persists. Is their metabolic effect on accumulation of liver fat the same or differs? Moreover, in real life they are not consumed separately but either as sucrose or high-fructose corn syrup. Data from studies concerned with acute changes in liver fat seem to be in support of the idea that, at least in healthy insulin sensitive subjects, fructose and not glucose plays an important role in accumulation of fat in the liver.

That is likely due to the fact that, contrary to glucose, almost all fructose is completely metabolized in the liver and its metabolism is not under feedback control at the level of fructokinase.

As the effects of dietary protein on liver fat accumulation are concerned, so far there is not enough data available to objectively evaluate its role.

Last but not least, the results of studies of acute changes of liver fat can explain relatively high intra-individual variability of HFC that was found to range between 15 to 20 % [66,67].

To summarize, studies of the effects of administration of selected nutrients on changes in liver fat content may provide valuable insights into mechanisms driving fat accumulation in the liver. The findings from these studies may directly translate into dietary recommendations and lifestyle changes that would prevent accumulation of fat in the liver and in this way contribute to the prevention of MASLD.

## Figures and Tables

**Table 1:** Overview of the studies of acute effects of nutrient administration on liver fat

Subjects	Design	Experimental meal	Change of liver fat (% of baseline)	
Szczepaniak 2004 [26]				
8 subjects age: ? BMI: ? HFC: <2.5 % (n=7) ~15 % (n=1)	liver fat measured before and 4 hours after mixed meal	mixed meal: 50 g fat/? g CHO/? g protein	no change	
Lindeboom 2015 [63]				
6 men, 3 women age: 23 ± 3 years BMI: 21.8 ± 1.8 kg/m^2^ HFC: 3.0 ± 0.7 g/kg ww	liver fat measured before, 3 and 5 hours after HF or HFP meal	high-fat mixed meal: 50 % of recomended daily caloric intake (1170 ± 180 kcal) 80 g fat/92 g CHO/20 g protein	+19 % at 3 hours, p<0.01 +18 % at 5 hours, p<0.01	no difference in the response of liver fat to high-fat and high-fat + protein diet (p = 0.84)
		high-fat high-protein mixed meal: high-fat + protein (1360 ± 210 kcal) 80 g fat/92 g CHO/71 g protein	+27 % at 3 hours, p<0.01 +31 % at 5 hours, p<0.01	
Bilet 2015 [64]				
10 overweight men age: 58 ± 8 years BMI: 28.7 ± 1.8 kg/m^2^ HFC: 2.7 ± 1.1 % 11 overweight men with MASLD age: 52 ± 5 years BMI: 30.4 ± 2.3 kg/m^2^ HFC: 13.1 ± 6.7 %	liver fat measured before 2-hour cycling exercise at 50 % of predetermined power output, 0.5 and 4 hours after exercise	glucose supplementation (20 % solution, 1.4 g/kg of weight) before exercise	no change	no difference in the response of both groups
		water in the same volume before exercise	+22 % at 4 hours post-exercise, p = 0.01	no difference in the response of both groups
Hernández 2017 [65]				
14 men age: 26 ± 5 years BMI: 22.5 ± 1.1 kg/m^2^ HF: 1.0 ± 0.7 %	liver fat measured 2 hours before and 4 hours after drink	drink containing palm oil 80 or 92 g fat (≈1.18 g/kg)	+35% at 4 hours	
		water	no change	
Dusilová 2019^a^ [66], Kovář 2021^b^ [67]				
^a^ 10 non-obese men age: 39 ± 10 years BMI: 26.9 ± 2.7 kg/m^2^ HF: 1.9 ± 1.0 % ^b^ 8 non-obese men with MASLD age: 40 ± 8 years BMI: 27.2 ± 2.2 kg/m^2^ HFC: 12.8 ± 7.3 %	liver fat measured before, at 3 and 6 hours	fat load: – heavy cream at time 0 150 g fat/15 g CHO/10 g protein	^a^ +19 % at 6 hours, p<0.05 ^b^ +10 % at 6 hours, p<0.01	
		fat load + fructose: – heavy cream at time 0 + 3 x 50 g of fructose at 0, 2, and 4 hours	^a^ +17 % at 6 hours, p<0.01 ^b^ +15 % at 6 hours, p<0.05	
		fat load + glucose: – heavy cream at time 0 + 3 x 50 g of glucose at 0, 2, and 4 hours	^a^ no change ^b^ +7 % at 3 hours, p<0.05, and returned to baseline at 6 hours	
		fructose: – 3 x 50 g of fructose at 0, 2, and 4 hours	^a^,^b^ no change	
		glucose: – 3 x 50 g of glucose at 0, 2, and 4 hours	^a^ −15 at 6 hours, p<0.05 ^b^ no change	
		prolonged fasting	^a^,^b^ no change	
Dusilová 2024 [69]				
16 obese men age: 47 ± 12 years BMI: 36.2 ± 3.8 kg/m^2^ HFC: 13.3 ± 8.5 %		fructose: – 3 x 50 g of fructose at 0, 2, and 4 hours	no change	
		glucose: – 3 x 50 g of glucose at 0, 2, and 4 hours	no change	
		prolonged fasting	no change	

CHO - carbohydrates, HFC - hepatic fat content
